# Limited detection of small (≤ 10 mm) colorectal liver metastasis at preoperative CT in patients undergoing liver resection

**DOI:** 10.1371/journal.pone.0189797

**Published:** 2017-12-15

**Authors:** Yousun Ko, Jihang Kim, Joseph Kyu-Hyung Park, Haeryoung Kim, Jai Young Cho, Sung-Bum Kang, Soyeon Ahn, Kyong Joon Lee, Kyoung Ho Lee

**Affiliations:** 1 Department of Radiology, Seoul National University Bundang Hospital, Gyeonggi-do, Korea; 2 Program in Biomedical Radiation Sciences, Department of Transdisciplinary Studies, Graduate School of Convergence Science and Technology, Seoul National University, Seoul, Korea; 3 Seoul National University Hospital, Seoul, Korea; 4 Department of Pathology, Seoul National University Hospital, Seoul National University College of Medicine, Seoul, Korea; 5 Department of Surgery, Seoul National University Bundang Hospital, Gyeonggi-do, Korea; 6 Division of Statistics, Medical Research Collaborating Center, Seoul National University Bundang Hospital, Gyeonggi-do, Korea; University Hospital Oldenburg, GERMANY

## Abstract

**Objective:**

To retrospectively determine the sensitivity of preoperative CT in the detection of small (≤ 10 mm) colorectal liver metastasis (CRLM) nodules in patients undergoing liver resection.

**Methods:**

The institutional review board approved the study and waived informed consent. We included 461 pathologically confirmed CRLM nodules in 211 patients (including 71 women; mean age, 66.4 years) who underwent 229 liver resections following abdominal CT. Prior to 163 resections, gadoxetic acid-enhanced liver MR imaging was also performed. Nodules were matched between pathology reports and prospective CT reports following a predefined algorithm. Per-nodule sensitivity of CT was calculated by nodule-size category. Generalized estimating equations were used to adjust for within-case correlation.

**Results:**

Fourteen nodule sizes were missing in the pathology report. Nodules of 1–5 mm and 6–10 mm accounted for 8.1% (n = 36) and 23.5% (n = 105) of the remaining 447 nodules, and the number of nodules gradually decreased as nodule size increased beyond 10 mm. The overall sensitivity of CT was 81.2% (95% confidence interval, 77.1%, 85.2%; 365/461). The sensitivity was 8% (0%, 17%; 3/36), 55% (45%, 65%; 59/105), 91%, 95%, and 100% for nodules of 1–5 mm, 6–10 mm, 11–15 mm, 16–20 mm, and >20 mm, respectively. The nodule-size distribution was similar between resections undergoing gadoxetic acid-enhanced MR imaging and those not undergoing the MR imaging.

**Conclusion:**

CT has limited sensitivity for nodules of ≤ 10 mm and particularly of ≤ 5 mm.

## Introduction

The overall survival rate in patients with colorectal liver metastasis (CRLM) is increased by the surgical removal of metastatic nodules in the liver [1, 2]. To ensure complete removal of CRLM nodules, a sensitive preoperative imaging test is required [3, 4]. CT plays an important role in the selection of patients for liver resection and in the localization of CRLM nodules [5].

Smaller CRLM nodules are of particular diagnostic concern, as they are more difficult to detect than larger nodules with preoperative and intraoperative examinations. Once detected, however, they are typically more amenable to surgical resection or local ablation. The reported sensitivity of CT in the detection of small (≤ 10 mm) CRLM nodules has ranged from 22% to 68% in previous studies [6]. This wide variation can be attributed to the differences in study methods and to the limited precision of individual studies due to the small numbers of nodules analyzed.

While CT is the primary imaging modality for preoperative staging and post-treatment surveillance, meta-analyses [6–8] have reported that magnetic resonance (MR) imaging is more sensitive than CT in the detection of CRLM. In detecting small (≤ 10 mm) malignant hepatic nodules, promising results have been reported particularly by using a hepatocyte-specific MR contrast agent (gadoxetic acid disodium; Primovist, Bayer Healthcare, Germany) [9, 10]. Such results have led radiology communities to suggest consensus guidelines recommending gadoxetic acid-enhanced MR imaging for preoperative evaluation of CRLM [11–13].

The purpose of our study was to precisely determine the sensitivity of preoperative CT in the detection of small (≤ 10 mm) CRLM nodules in patients undergoing liver resection by using pathological findings as the reference standard.

## Materials and methods

### Study overview

The institutional review board of Seoul National University Bundang Hospital approved this retrospective study and waived the requirement for informed consent. All data were fully anonymized and aggregated prior to analysis. We retrospectively included 211 patients who underwent 229 liver resections for CRLM following preoperative CT. We analyzed the size distribution of the CRLM nodules identified in pathological examination, and calculated the per-nodule sensitivity of CT for each nodule-size category.

### Study sample

**Fig 1:**
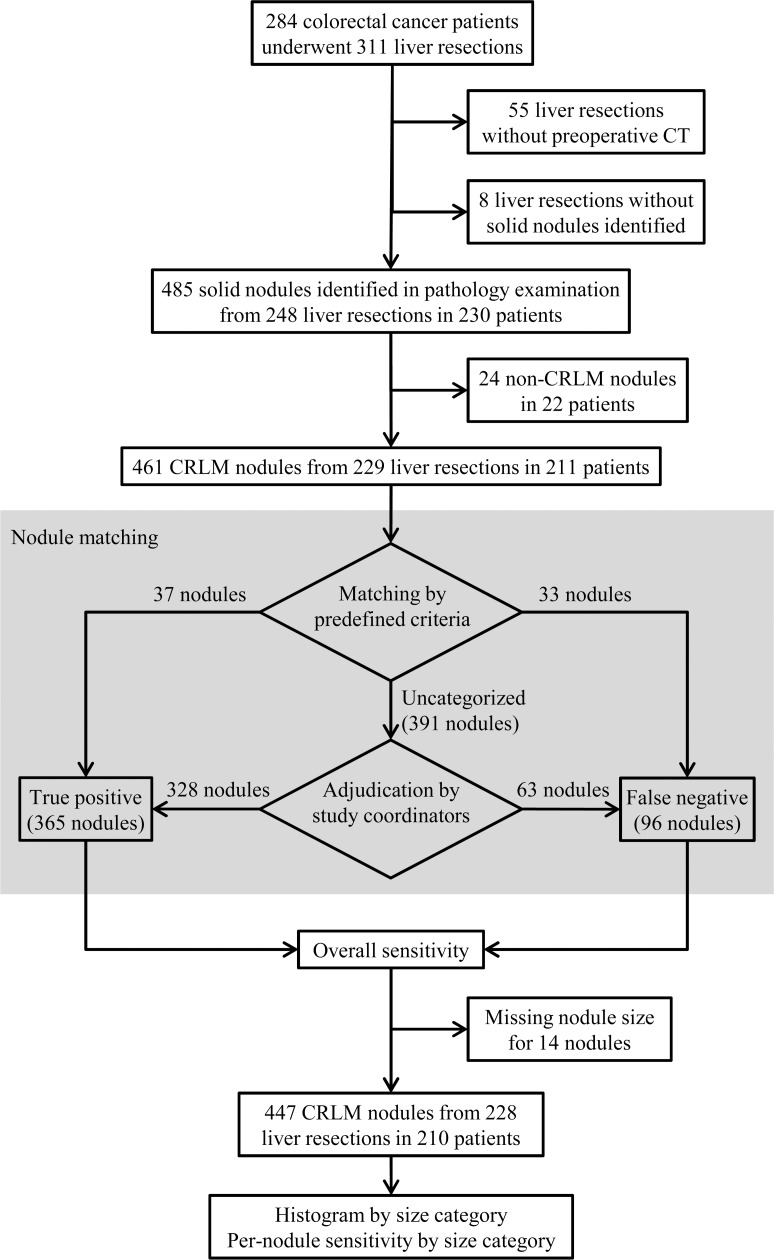
Patient flow diagram. CRLM = colorectal liver metastasis.

From the surgical database of Seoul National University Bundang Hospital, a teaching hospital in Korea, we identified 284 patients who had pathologically-confirmed colorectal cancer and underwent 311 liver resections from 2003 through 2014. We excluded 55 liver resections without preoperative contrast-enhanced CT within 30 days before liver resection, and eight liver resections from which no solid nodule was identified on pathology examination. In the remaining 248 liver resections performed in 230 patients, the pathological examination revealed 485 solid hepatic nodules. We additionally excluded 24 pathologically confirmed non-CRLM nodules in 22 patients, including hepatocellular carcinoma (n = 9), cholangiocarcinoma (n = 6), non-neoplastic lesions (n = 5), hemangioma (n = 3), and metastasis from gallbladder cancer (n = 1). The study finally included 461 pathologically confirmed CRLM nodules in 211 patients who underwent 229 liver resections (Fig 1 and Tables 1 and 2). The 211 patients (mean age ± standard deviation, 66.4 ± 10.9 years) comprised 140 men (67.5 ± 10.7 years) and 71 women (64.2 ± 11.2 years). Among these patients, 16 patients underwent liver resection twice and a single patient underwent liver resection three times.

**Table 1:** Characteristics of the 211 patients who underwent liver resection for colorectal liver metastasis following abdominal CT.

Patient characteristics		Data
Age (y)^a^		
	Total	66.4 ± 10.9
	Female	64.2 ± 11.2
	Male	67.5 ± 10.7
Sex		
	Female	71 (33.6%)
	Male	140 (66.4%)
Body mass index (kg/m^2^)^a^^b^		23.7 ± 3.4
Diffuse liver disease		
	None	202 (95.7%)
	Hepatitis B	7 (3.3%)
	Hepatitis C	1 (0.5%)
	Alcoholic steatohepatitis	1 (0.5%)
	Nonalcoholic steatohepatitis	0 (0%)
	Cholangitis	0 (0%)
T stage of primary colorectal cancer		
	pT1	2 (0.9%)
	pT2	12 (5.7%)
	pT3	142 (67.3%)
	pT4	46 (21.8%)
	pTx	9 (4.3%)
N stage of primary colorectal cancer		
	pN0	34 (16.1%)
	pN1	79 (37.4%)
	pN2	90 (42.7%)
	pNx	8 (3.8%)

^a^Data are mean (and standard deviation). Otherwise, data are numbers of patients (and the percentages).

^b^Weight in kilograms divided by the square of the height in meters.

**Table 2:** Characteristics of the 229 liver resections included in the study.

Liver resection				Data
Study period				
	2003–2005			20 (8.7%)
	2006–2008			60 (26.2%)
	2009–2011			78 (34.1%)
	2012–2014			71 (31.0%)
Primary colorectal cancer				
	Concurrent resection			140 (61.1%)
	Past resection			89 (38.9%)
Chemotherapy before liver resection				90 (39.3%)
Interval between preoperative CT and liver resection (days)^a^				9 (5–19)
Additional liver MR imaging				
	Not performed			25 (10.9%)
	Performed			204 (89.1%)
		Contrast agent		
			Gadodiamide	2 (1.0%)
			Ferucabotran	39 (19.1%)
			Gadoxetic acid disodium	163 (79.9%)
		Magnet		
			1.5-T	165 (80.9%)
			3.0-T	39 (19.1%)
Surgical approach				
	Laparotomy			182 (79.5%)
	Laparoscopy			47 (20.5%)
Concurrent intraoperative radiofrequency ablation in the remnant liver				36 (15.7%)
Number of nodules per resection				
	1			129 (56.3%)
	2			47 (20.5%)
	3			18 (7.9%)
	4			15 (6.6%)
	5–10			20 (8.7%)

MR = magnetic resonance

^a^Data are median (and interquartile range). Otherwise, data are numbers of patients (and the percentages).

Ten of the 461 CRLM nodules included in this study have been included in another retrospective study that investigated the value of gadoxetic acid-enhanced MR imaging in assessing indeterminate hepatic focal lesions at preoperative CT [14].

### CT imaging and reporting

Intravenous contrast-enhanced CT images were obtained during the portal venous phase using 16- or higher detector-row machines (S1 Table). From each helical scan, transverse images were reconstructed with a thickness of 4 or 5 mm and an increment of 3 or 4 mm using a standard filtered back projection. Coronal images were reformatted with the same thickness and increment.

Five abdominal radiologists with 3–13 years of experience made the CT reports prospectively as part of their daily practice. Narrow window settings (typically with the window width and level of 200 and 100 Hounsfield units, respectively) were used to evaluate the liver. The radiologists recorded the location (Couinaud segment) and size (maximum transverse diameter in millimeters) of each suspected CRLM nodule in the CT reports according to a standardized structured report form. Throughout the study period, we adhered to the strict policy of using the structured report form for preoperative CT in patients with colorectal cancer to standardize the communication among radiologists, surgeons, and pathologists.

### MR imaging

For 204 liver resections in 186 of the 211 patients, preoperative liver MR imaging was performed following CT. While no clear guidelines were available regarding the use of liver MR imaging in patients with colorectal cancer during the study period, our surgeons and physicians generally added MR imaging if CT showed potentially resectable CRLM [5] or indeterminate focal hepatic lesions that could not be characterized with CT.

Due to the installation of new MR equipment and the introduction of new contrast agents, our MR techniques changed during the study period. We used 1.5-T magnets during the initial part of the study, and 3-T magnets during the later part of the study (Philips Healthcare, The Netherlands). The contrast agent used changed from gadodiamide (Omniscan; GE Healthcare, Princeton, NJ) (n = 2) to ferucarbotran (Resovist; Schering, Berlin, Germany) (n = 39), and eventually to gadoxetic acid disodium (n = 163). Imaging generally consisted of dual-echo in- and opposed-phase spoiled gradient-echo T1-weighted, fat-suppressed fast spin-echo T2-weighted, diffusion-weighted, and dynamic fat-suppressed spoiled gradient-echo T1-weighted imaging. The abdominal radiologists interpreted the images.

### Liver resection

The need for liver resection for each patient was determined at a weekly multidisciplinary conference involving surgeons, oncologists, radiologists, and pathologists. During surgery, the surgeons with 3–17 years of experience mobilized and evaluated the liver by inspection and/or palpation. In addition, the surgeons or radiologists who had full knowledge of the preoperative imaging findings performed intraoperative liver ultrasonography (SSD-3500, Aloka, Japan; MyLab 25 Gold, Esaote Biomedica, Italy; or iU22, Philips Medical Systems, The Netherlands).

### Pathological examination

Pathological examinations were performed by one of two pathologists with 3–12 years of experience. In cutting liver specimens for gross examination, it was our internal guideline to keep the tissue slice as 5 mm or thinner, although a tissue thickness up to 10 mm is generally accepted [15]. For each slice, a gross photograph was obtained. The pathologists identified solid nodules by careful macroscopic inspection of the liver slices. All suspected nodules were sampled, embedded in paraffin blocks, and stained with hematoxylin-eosin for microscopic examination. For each nodule, the pathologist recorded the location in terms of the Couinaud segment and the nodule size in millimeters in the pathology reports. This information served as the reference standard for the subsequent analyses. Hereafter, the size of a given nodule refers to the size recorded in the pathology report.

### Nodule-matching algorithm

For a pathologically identified nodule to be regarded as a true positive, the same nodule had to be documented in the prospective CT report. Any pathologically confirmed CRLM nodule that was not documented in the CT reports was categorized as a false negative. False-positive nodules were not counted because our retrospectively collected study sample included only pathologically confirmed CRLM nodules.

Two study coordinators (radiologists with clinical experience of 13 and 4 years), who did not participate in making CT or MR reports, conducted the matching of nodules between the pathology reports and the CT reports following a predefined algorithm. First, the study coordinators together reviewed the CT reports and pathology reports to select true-positive nodules by using the following predefined criteria. For a pathologically identified nodule to be regarded as a true positive, the pathological report and CT report had to be concordant for both the segmental location and the size of the nodule. For nodule size, an acceptable margin of error was empirically set as 3 mm for small (≤ 10 mm) nodules and 5 mm for larger (≥ 10 mm) nodules. If two or more nodules were found in the same liver segment, the larger nodule(s) was matched preferentially based on the assumption that larger nodules were more detectable at both CT and pathological examination than smaller nodules. Second, for nodules that were pathologically identified but left uncategorized as true positive or false negative in the first step of the nodule matching, the study coordinators in consensus adjudicated the nodule matching. For the adjudication to link the pathological reports with the CT reports, the study coordinators reviewed all available CT and MR images, specimen photographs, and surgical records.

### Statistical analysis

Two radiologists and a statistician planned all analyses before the data collection. A histogram of the nodule-size distribution was created by categorizing nodule size by 5-mm increments. The per-nodule CT sensitivity was calculated for each size category. The population-averaged sensitivity estimates and their 95% confidence intervals (CIs) were adjusted for correlations within a liver resection case by using the generalized estimating equations approach across size-categorized subgroups [16]. We used an intercept-only model and assumed an exchangeable working correlation structure. Logistic regression with a logit link was used for the binary outcome (i.e., sensitivity). A Chi-square test was performed to identify the difference in the 1-year recurrence rate of CRLM nodules between the liver resections with and without known false negatives. Statistical analyses were performed using Stata version 14.0 software package (StataCorp, College Station, TX).

Additionally, the same analyses were performed separately for the liver resections following preoperative gadoxetic acid-enhanced MR imaging and for the resections without preoperative gadoxetic acid-enhanced MR imaging. We did not formally compare these two subgroups because such a comparison would be confounded by many factors including the indications for MR imaging, MR equipment, and institutional experience, all of which were affected by the study period. We did not compare the sensitivity between CT and MR imaging, since CT preceded MR imaging in each patient and the two studies were not read independently. The diagnostic yield (in terms of the number of CRLM nodules) of additional gadoxetic acid-enhanced MR imaging in our center has been previously reported [14].

## Results

### Nodule size

**Fig 2:**
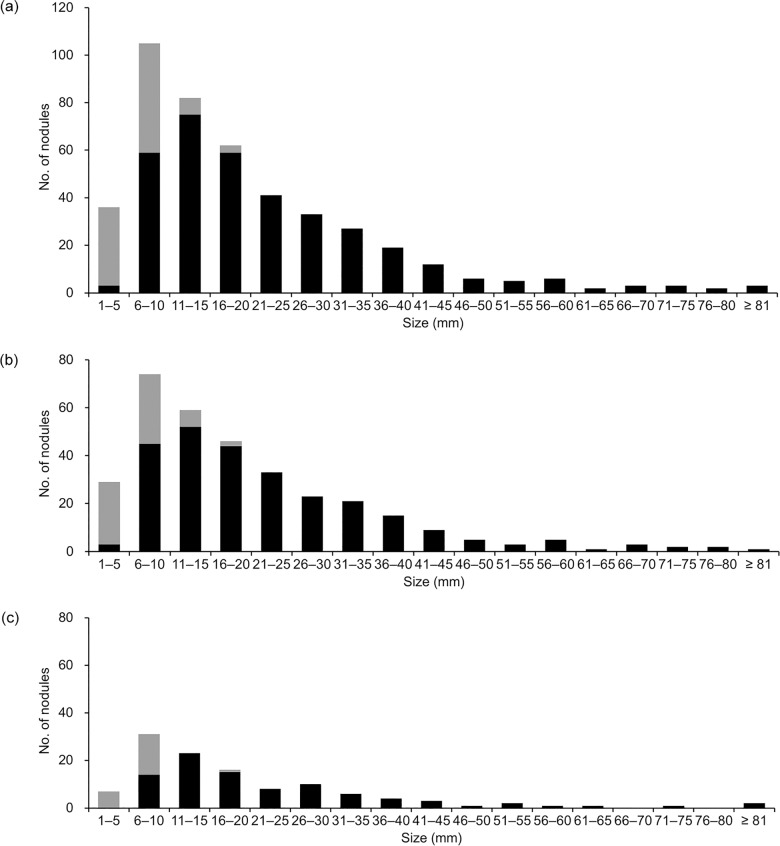
Histograms of size distribution of pathologically confirmed colorectal liver metastasis nodules. Nodule size refers to that recorded in pathology reports. Dark and gray bars represent nodules detected (true positives) and undetected (false negatives) at preoperative CT, respectively. Fourteen nodules were not included in the histograms as their sizes were missing in the pathology reports. (a) 447 nodules from 228 liver resections. (b) Subgroup of 331 nodules from 163 resections following gadoxetic acid-enhanced MR imaging. (c) Subgroup of 116 nodules from 65 resections without gadoxetic acid-enhanced MR imaging.

From the 229 liver resections in the 211 patients, 461 CRLM nodules were documented in the pathology reports. The number of nodules per liver resection ranged from one to 10. For 14 nodules, the size was missing in the pathology reports. The remaining 447 nodules from 228 liver resections in 210 patients were included in the histogram (Fig 2, S2 Table). Nodules of 1–5 mm and 6–10 mm accounted for 8.1% (n = 36) and 23.5% (n = 105) of the 447 nodules, respectively. For nodules of 6–10 mm or larger, the number of nodules in a size category gradually decreased as the nodule size increased.

### Nodule matching

With the first-step matching using predefined criteria, 37 and 33 of the 461 nodules were judged to be true positives and false negatives, respectively. The remaining 391 nodules required the second-step adjudication by the study coordinators, who determined 328 and 63 nodules as true positives and false negatives, respectively (Fig 1). Therefore, 365 and 96 nodules were finally determined as true positives and false negatives, respectively.

### Sensitivity of CT

**Fig 3:**
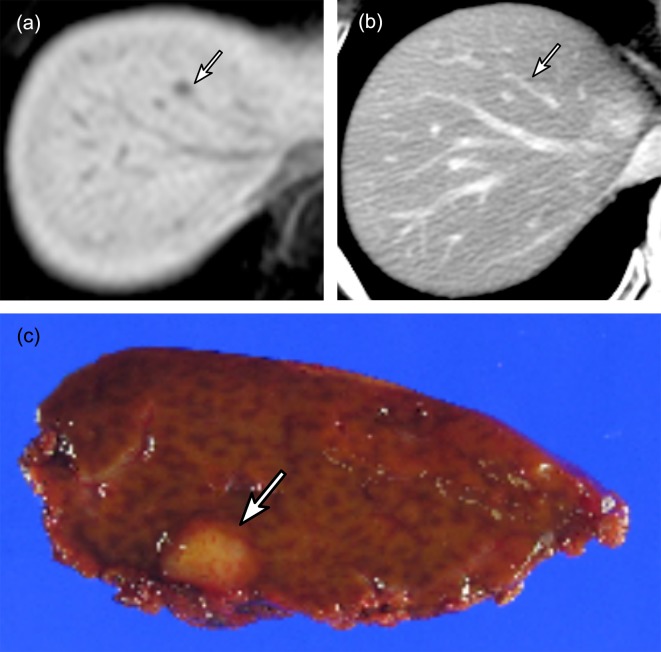
A 64-year-old woman with colorectal liver metastasis (CRLM). (a) Gadoxetic acid-enhanced transverse T1-weighted MR image during the hepatobiliary phase shows a small nodule (arrow) in segment 4. (b) The nodule (arrow) is not clearly seen at contrast-enhanced transverse CT image. (c) The nodule (arrow) measured 5 mm and was confirmed to be CRLM.

The overall sensitivity of CT for the pathologically confirmed CRLM nodules was 81.2% (95% CI, 77.1%, 85.2%; 365/461). There were 96 false-negative nodules from 65 liver resections in 65 patients (Figs 2 and 3). One false-negative nodule occurred in 46 resections, two occurred in 13 resections, and three or more occurred in six resections. Of these false-negative nodules, 33 were 1–5 mm in size, 46 were 6–10 mm, seven were 11–15 mm, and three were 16–20 mm, and in seven nodules the size was missing. There were no false-negative nodules of 21–25 mm or larger. Among the 65 resections with false-negative nodules, true-positive nodules were confirmed in 51 resections.

The 14 nodules (including the seven false-negative nodules) with missing size information in the pathology reports were not included in the following sensitivity calculations by size category. The sensitivity of CT increased as the nodule size increased. For the nodules of 1–5 mm and 6–10 mm, the sensitivities were 8% (0%, 17%; 3/36) and 55% (45%, 65%; 59/105), respectively. When the two size categories were combined, the sensitivity for the nodules of ≤ 10 mm was 43% (34%, 53%; 62/141). For the nodules of 11–15 mm and 16–20 mm, the sensitivities were 91% (84%, 98%, 75/82) and 95% (90%, 100%, 59/62), respectively. The sensitivity reached 100% (162/162) for nodules of 21–25 mm or larger. The sensitivities for each size category are shown in Fig 2 and the cumulative sensitivities for different nodule-size thresholds are shown in Table 3.

**Table 3:** Cumulative per-nodule sensitivities of CT for different nodule-size thresholds.

Size category	229 liver resections	163 liver resections following gadoxetic acid-enhanced MR imaging	66 liver resections without gadoxetic acid-enhanced MR imaging
All nodules including those with missing size information	81.2% (77.1%, 85.2%) [365/461]	82.9% (78.3%, 87.5%) [274/341]	77% (68%, 85%) [91/120]
All nodules with available size information	81.9% (78.0%, 85.8%) [358/447]	83.5% (79.0%, 88.0%) [267/331]	78% (71%, 85%) [91/116]
> 5 mm	86.5% (83.1%, 90.0%) [355/411]	87.9% (84.0%, 91.9%) [264/302]	83% (78%, 89%) [91/109]
> 10 mm	96.9% (94.7%, 99.1%) [296/306]	96.2% (93.5%, 99.0%) [219/228]	99% (96%, 100%) [77/78]
> 15 mm	98.7% (97.2%, 100.0%) [221/224]	99% (97%, 100%) [167/169]	98% (95%, 100%) [54/55]
> 20 mm	100% (NA) [162/162]	100% (NA) [123/123]	100% (NA) [39/39]

MR = magnetic resonance, NA = could not be calculated. Nodule size refers to that recorded in pathology report. Sensitivities (and the 95% confidence intervals) were adjusted for within-case correlation. Data in brackets are the number of nodules used for the calculation of the sensitivities.

There was statistically significant difference (p = 0.048) in the 1-year recurrence rate of CRLM nodules between the liver resections with known false negatives (34%; 22/65) and the resections where the preoperative CT successfully detected all CRLM nodules found on pathological examination (21%; 35/164).

### Subgroup analysis

Histograms were drawn separately for 331 nodules from 163 liver resections following preoperative gadoxetic acid-enhanced MR imaging (in 149 patients) and for the 116 nodules from the 66 resections without preoperative gadoxetic acid-enhanced MR imaging (in 62 patients). We did not observe any notable difference between the two subgroups in the overall nodule-size distribution or the sensitivity of CT according to size category (Fig 2, Table 3).

## Discussion

In our results, the sensitivity of CT was limited to 8% for CRLM nodules of 1–5 mm and 55% for nodules of 6–10 mm. When the two size categories were combined, the sensitivity for nodules of ≤ 10 mm was 43%. The sensitivity increased with increasing nodule size, reaching 100% for nodules of 21–25 mm or larger.

Nodules of 1–5 mm were rarely identified in our pathological examination, accounting for only 8.1% (n = 36) of the 447 CRLM nodules with available size records. On the contrary, nodules of 6–10 mm accounted for 23.5% (n = 105). Beyond 10 mm, the number of nodules in a size category then gradually decreased as nodule size increased. By extrapolating the overall trend of the histogram to the size category of ≤ 5 mm, we postulate that our study sample may have failed to include a considerable number of small (particularly of ≤ 5 mm) CRLM nodules since they were undetected with preoperative imaging studies and ordinary pathological examination. This also implies that the true sensitivity of CT may be lower than the results of this kind of investigations.

As previously noted, previous studies reported a wide range of sensitivity of CT for the detection of small (≤ 10 mm) CRLM nodules [6]. As compared with the previous studies, our study has the following distinctive features toward conservative estimation of sensitivities. First, our study sample included a large number of small (≤ 10 mm) CRLM nodules (n = 141). Previous studies had limited precision in measuring the sensitivity due to the limited numbers of small (≤ 10 mm) CRLM nodules included in the individual studies. To our knowledge, the two largest previous series regarding CRLM nodules of ≤ 10 mm included 65 and 47 nodules, with reported sensitivities of 48% and 38%, respectively [17, 18].

Second, only a limited number of previous studies [19, 20] have used pathologic confirmation as the sole reference standard as we did in our study. Other studies used imaging follow up as well as pathologic confirmation as the reference standard, which may have inflated the sensitivity of CT particularly for small nodules due to the failure to include some tiny CRLM nodules that existed but could not be visualized in the initial imaging studies. For example, a recent prospective study [21] using imaging follow up as the reference standard reported the sensitivity of CT as 58% for 28 small (≤ 10 mm) nodules, which can be compared with the corresponding sensitivity of 43% in our results. In addition, the size measurement at CT is unlikely reproducible for small (≤ 10 mm) nodules as many of those nodules have indistinct margins.

Third, we retrospectively analyzed prospective CT reports in a cohort of consecutive CRLM resection cases. We chose this study design to represent a clinical practice. In some previous studies, a very small number of radiologists retrospectively reviewed images in a full-factorial design [22, 23], and it was often unclear if the involved radiologists were blinded to the prevalence of CRLM in the study samples or to the patient inclusion criteria of confirmed or suspected CRLM [23, 24].

Finally yet importantly, we clarified the nodule-matching algorithm between the pathology and CT findings. Our first-step matching with predefined criteria yielded only a small number of true-positive (n = 37) and false-negative (n = 33) nodules, and left many nodules uncategorized (n = 391). This finding clearly shows that individual nodule matching is frequently not straightforward, and therefore inevitably involves investigators’ subjective opinion to some extent [9, 25], as in our second-step adjudication. Surprisingly, previous studies on CRLM have rarely addressed the difficulties in nodule matching, the associated study limitations, or the measures required to overcome those limitations. For example, we were unable to find any study that clarified the acceptable margin of error for nodule-size matching. In the investigations on malignant hepatic nodules (not limited to CRLM), several researchers have attempted rigorous nodule matching by using advanced techniques. These techniques included using *ex vivo* multiplanar reformation MR images as a link between preoperative cross-sectional images and pathological sections [9], using infusion fixation of liver specimens [9, 26], cutting liver specimens in the approximate plane of preoperative imaging [27], and cutting liver specimens into very thin (e.g., 3 mm) slices [9, 26]. As expected, these studies reported low sensitivities of preoperative imaging for the detection of malignant hepatic nodules. We were unable to use those advanced techniques in our retrospective study. Further investigation is required to improve and validate the techniques and algorithms in nodule matching for future studies on the preoperative imaging diagnosis of malignant hepatic nodules.

Our subgroup analysis results should be interpreted cautiously. Although our results rather showed no notable difference between the two subgroups in the sensitivity and overall diagnostic yield (i.e., nodule number) according to size category, the comparability between the two subgroups is limited due to the confounders associated with study period. As suggested by previous studies [9, 10] on malignant hepatic nodules (not limited to CRLM), gadoxetic acid-enhanced MR imaging appears to detect additional nodules of 6–10 mm. However, this improvement in sensitivity is likely limited for larger nodules because the sensitivity of CT for larger lesions is already high. Furthermore, the sensitivity improvement for nodules of ≤ 5 mm also appears to be limited, because most of these tiny nodules are missed even with gadoxetic acid-enhanced MR imaging [9, 10]. Although a recent article noted that additional detection of hepatocellular carcinoma nodules with gadoxetic acid-enhanced MR imaging may reduce the risk of disease recurrence and overall mortality [28], there is sparsity of evidence that the detection of tiny CRLM nodules would enhance clinical outcomes. As the 1-year recurrence rate of CRLM nodules is significantly higher in liver resections with false-negative nodules on preoperative CT (p = 0.048), it can be inferred that higher sensitivity of CRLM nodules potentially lead to better outcomes. Therefore, further studies are required to prove if an increased sensitivity of gadoxetic acid-enhanced MR imaging for small (≤ 10 mm) malignant hepatic nodules [9, 29, 30] can translate into improved clinical outcomes in patients with suspected CRLM. It is also important to identify which subgroups of colorectal cancer patients would clinically benefit from the addition of gadoxetic acid-enhanced MR imaging. Also, it should be noted that eight liver resections were excluded because pathologic examinations failed to identify any solid nodules (Fig 1). It can be assumed that false-positive results on preoperative imaging could possibly lead to unnecessary surgical interventions.

Our study has limitations. First, our study sample may have failed to include some small (particularly of ≤ 5 mm) CRLM nodules that were undetected with preoperative imaging studies and therefore were not resected. Second, as previously stated, since the pathological specimens were cut with a thickness up to 5 mm, some tiny nodules smaller than the slice thickness may have been undetected in the pathological examination, and therefore failed to enter the study sample. Third, because of tissue shrinkage during the preparation for pathological examination [15], a nodule size measured in the pathological specimen was likely to be smaller than the actual size *in vivo* at the time of CT examination. Taking into consideration these study limitations, the true number of small CRLM nodules would be greater, and the true sensitivity of CT for small nodules would be even lower than what we observed.

In conclusion, CT has limited sensitivity for nodules of ≤ 10 mm and particularly for nodules of ≤ 5 mm, while the sensitivity reaches 100% for larger (> 20 mm) nodules. Further investigations are required to establish a more sensitive imaging study, such as gadoxetic acid-enhanced MR imaging, to supplement the limited sensitivity of CT and to improve the clinical outcomes in patients with suspected CRLM.

## Supporting information

S1 Table CT imaging parameters. (DOCX)

S2 Table Distribution of nodule size. Data represent the number of nodules.
(DOCX)
