# Supplementary material for: Limited detection of small (≤ 10 mm) colorectal liver metastasis at preoperative CT in patients undergoing liver resection
Source: PLoS One. 2017 Dec 15;12(12):e0189797. doi: 10.1371/journal.pone.0189797 (PMC5731738; doi:10.1371/journal.pone.0189797)
Supplement: S1 Table — (DOCX) [file pone.0189797.s001.docx]

# S1 Table. CT imaging parameters.

| **Protocol** | | **Machine** | | |
| --- | --- | --- | --- | --- |
|  |  | **16-detector-row** | **64-detector-row** | **256-detector-row** |
| Scan | |  |  |  |
|  | Range | From 4 cm above the liver dome to 1 cm below the ischial tuberosity | Same | Same |
|  | Collimation | 16 × 1.5 mm | 64 × 0.625 mm | 128 × 0.625 mm, double sampling |
|  | Rotation speed | 0.5 sec | Same | Same |
|  | Pitch | 1 | 0.89 | 0.99 |
|  | Tube potential (kVp) | 120 | 120 or 100 | 120 or 100 |
|  | Automatic tube current modulation | ACS combined with D-Dom | ACS combined with Z-Dom | ACS combined with Z-Dom |
|  | Reference tube-current–time product (mAs) | 180 | 260 for 120 kVp  or 380 for 100 kVp | 200 for 120 kVp  or 360 for 100 kVp |
| Reconstruction | |  |  |  |
|  | Reconstruction filter | Standard soft tissue | Same | Same |
|  | Thick transverse images | 5-mm thickness with 20% overlap | 4-mm thickness with 25% overlap | 4-mm thickness with 25% overlap |
|  | Thin transverse images | 2-mm thickness with 50% overlap | Same | Same |
|  | Additional coronal reformation | 5-mm thickness with 20% overlap | 4-mm thickness with 25% overlap | 4-mm thickness with 25% overlap |
