# Supplementary material for: Limited detection of small (≤ 10 mm) colorectal liver metastasis at preoperative CT in patients undergoing liver resection
Source: PLoS One. 2017 Dec 15;12(12):e0189797. doi: 10.1371/journal.pone.0189797 (PMC5731738; doi:10.1371/journal.pone.0189797)
Supplement: S2 Table — Data represent the number of nodules. (DOCX) [file pone.0189797.s002.docx]

# S2 Table. Distribution of nodule size. Data represent the number of nodules.

| **Size category (mm)** | **229 liver resections** | | **163 liver resections following gadoxetic acid-enhanced MR imaging** | | **66 liver resections without gadoxetic acid-enhanced MR imaging** | |
| --- | --- | --- | --- | --- | --- | --- |
|  | **True positive** | **False negative** | **True positive** | **False negative** | **True positive** | **False negative** |
| Missing size information | 7 | 7 | 7 | 3 | 0 | 4 |
| 1–5 | 3 | 33 | 3 | 26 | 0 | 7 |
| 6–10 | 59 | 46 | 45 | 29 | 14 | 17 |
| 11–15 | 75 | 7 | 52 | 7 | 23 | 0 |
| 16–20 | 59 | 3 | 44 | 2 | 15 | 1 |
| 21–25 | 41 | 0 | 33 | 0 | 8 | 0 |
| 26–30 | 33 | 0 | 23 | 0 | 10 | 0 |
| 31–35 | 27 | 0 | 21 | 0 | 6 | 0 |
| 36–40 | 19 | 0 | 15 | 0 | 4 | 0 |
| 41–45 | 12 | 0 | 9 | 0 | 3 | 0 |
| 46–50 | 6 | 0 | 5 | 0 | 1 | 0 |
| 51–55 | 5 | 0 | 3 | 0 | 2 | 0 |
| 56–60 | 6 | 0 | 5 | 0 | 1 | 0 |
| 61–65 | 2 | 0 | 1 | 0 | 1 | 0 |
| 66–70 | 3 | 0 | 3 | 0 | 0 | 0 |
| 71–75 | 3 | 0 | 2 | 0 | 1 | 0 |
| 76–80 | 2 | 0 | 2 | 0 | 0 | 0 |
| ≥ 81 | 3 | 0 | 1 | 0 | 2 | 0 |
